# Therapists’ emotional state after sessions in which suicidality is addressed: need for improved management of suicidal tendencies in patients with borderline personality pathology

**DOI:** 10.1186/s12888-021-03549-9

**Published:** 2021-11-23

**Authors:** Vera Bühlmann, Susanne Schlüter-Müller, Lukas Fürer, Martin Steppan, Marc Birkhölzer, Klaus Schmeck, Julian Koenig, Michael Kaess, Ronan Zimmermann

**Affiliations:** 1 Child and Adolescent Psychiatric Research Department, Psychiatric University Hospital, University of Basel, Basel, Switzerland; 2 Juvenile Forensic Department, Psychiatric University Hospital, University of Basel, Basel, Switzerland; 3 University Hospital of Child and Adolescent Psychiatry and Psychotherapy, University of Bern, Bern, Switzerland; 4 Department of Child and Adolescent Psychiatry, Center for Psychosocial Medicine, University Hospital Heidelberg, Heidelberg, Germany; 5 Department of Child and Adolescent Psychiatry, Psychosomatics and Psychotherapy, Faculty of Medicine and University Hospital Cologne, University of Cologne, Cologne, Germany; 6 Division of Clinical Psychology and Psychotherapy, Faculty of Psychology, University of Basel, Basel, Switzerland

**Keywords:** Psychotherapy, Countertransference, Adolescents, Suicidality, Stress, Borderline personality pathology

## Abstract

**Introduction:**

Patient suicidality is a frequently experienced topic for psychotherapists. Especially adolescents with borderline personality pathology (BPP) often exhibit suicidal tendencies. Previous research which examined therapists’ countertransference towards suicidal patients suggested that therapists are negatively affected and distressed by them. We hypothesize that this emotional response of the therapists is related to specific sessions in which suicidality came up as a topic. Accordingly, the objective of this study consists in examining therapists’ emotional state on a session level of analysis.

**Methods:**

The sample consisted of *N* = 21 adolescents (age 13–19 years) with BPD or subthreshold BPD. Therapists’ emotional states were measured in *n* = 418 sessions using the Session Evaluation Questionnaire. Principal component analysis was used to reduce dimensionality of the therapist response. The emotional states were compared depending on whether suicidality has been addressed in the session (SS) or not (NSS).

**Results:**

Two components could be identified. Firstly, therapists were more aroused, excited, afraid, angry and uncertain after SS than after NSS. Secondly, therapists were more aroused, excited, definite and pleased after SS than after NSS. *Discussion:* Suicidality does not always have to be a burden for therapists: Both a “distress” and an “eustress” component occur in this context from which the latter is supposed to help clinicians master a difficult situation. Since countertransference feelings are often not fully conscious, it is necessary to do research on therapists’ emotional states after sessions in which suicidality is addressed. This is crucial to both prevent the therapeutic process from being endangered and preserve clinicians’ mental health. Clinical implications and limitations are discussed.

## Introduction

Suicidality is defined as “the risk of suicide, usually indicated by suicidal ideation or intent, especially as evident in the presence of a well-elaborated suicidal plan” [1]. 50 to 95% of the clinicians report having experienced patients with some form of suicidal ideation or behavior [2–4]. Moreover, suicidal statements [5] and suicidal ideation [6] were considered as one of the most stressful aspect of the therapeutic work. The current study investigates the impact of suicidality addressed in psychotherapeutic sessions on therapists’ post-session emotional state.

Suicidality plays an important role in Borderline Personality Disorder (BPD). 60 to 70% of patients with BPD commit a suicide attempt at some point in their life and the rate of completed suicide is 50 times higher in patients with BPD than in the general population [7, 8]. Despite the controversy regarding the diagnosis of BPD in underage patients, there is convincing evidence that diagnosing BPD in adolescents is as reliable and valid as in adults [9]. The elimination of the age limit of previous versions for the diagnosis of personality disorders in the International Classification of Diseases 11th revision (ICD-11 [10];) and the consequent possibility of early diagnosis provides the opportunity for effective early intervention [11]. Thus, a chronification and long-lasting impact of the personality disorder could be prevented.

The term countertransference is of great historical importance for therapeutic work since it has heightened therapists’ awareness of their own feelings and has been coined in different ways. In the last decades, it has received more attention since it was acknowledged having great impact on treatment process and outcome [2, 12]. The totalistic view defines countertransference as the total emotional reaction of the psychotherapist to the patient in the treatment situation [13]. The term countertransference is important in that it provides a historical framework for this question, but for the sake of simplicity and conceptual clarification, the following study will refer to “therapist’s emotional response” or the operationalized form of it, “emotional state”.

In association with suicidal patients, the term “countertransference hate” was introduced [14], which can arise in psychotherapists when working with these clients. Its management through full awareness is essential for therapy success, since therapists’ emotional response is assumed to predict patients’ suicide outcome [15]. Moreover, the therapist’s fear of losing a patient to suicide impedes an adequate assessment of the patient’s inner experience, leads to countertransference reactions and thus to alliance ruptures [16, 17]. The fear that a client could commit suicide represents a widespread therapeutic feeling [18] and interferes with their ability to work [19]. Therefore, it is crucial to conduct research on therapists’ emotional states after psychotherapy sessions in which suicidality is an issue.

Scientific evidence about therapist’ countertransference towards suicidal patients is broadly consistent and suggest that it involves negative emotions such as anxiety, anger and higher self-reported distress [3, 5, 6, 14]. Therapist stress is related to increased feelings of anxiety, tension, hopelessness, fear or embarrassment [20]. Others assume a wider spectrum of reactions ranging from hopelessness or sense of failure to the desire to intrusively nurture the patient [21]. A recent systematic review which included ten quantitative studies examined health care professionals’ countertransference toward suicidal patients [22]. Results showed that “suicidal patients elicit disinterest, anxiety, confusion, overwhelming, entrapment, rejection, inadequacy, helplessness or distress – but also engangement and fulfillment – among healthcare professionals, which suggests a specific and mostly adverse suicidal-related countertransference.” (p. 10). Moreover, the authors draw the conclusion that current suicidal ideation seems to be involved in eliciting such countertransference [12, 23], whereas the evidence on current or past suicidal behavior is inconclusive [12, 24–26]. Nevertheless, it should be stated that study designs, settings, measurement methods of countertransference and suicidality varied widely across the included studies, making any quantitative synthesis of findings difficult.

Since previous research used a between-subject design comparing therapist responses towards suicidal versus towards non-suicidal patients, the question remains whether specific suicidal statements or general personality structure mediated the correlation with therapist response. Moreover, research has mostly focused on therapist traits rather than states such as affect. Therefore, it is important to examine within-therapist variables to further the understanding of therapist factors [27]. The current study meets the need for a prospective study design that can reveal differences between sessions within the patient-therapist dyad. In this way, it can be investigated what influence actual suicidal expressions have on clinicians. Our hypothesis is that suicidality addressed in a psychotherapy session burdens the therapist in terms of more negative emotions and distress measured after the session. The aim of this study is to discover a pattern of therapist’ emotional state after sessions in which suicidality was addressed. This awareness of the emotional state after specific sessions allows for a concrete intervention and prevention to ensure both therapists’ and patients’ mental health.

## Methods

Some of the method passages are adopted from Zimmermann et al. [28, 29] where the study design has been described in more detail.

### Ethics approval and consent to participate

All methods were carried out in accordance with relevant guidelines and regulations. All experimental protocols were approved by the ‘Ethikkommission Nordwest- und Zentralschweiz’. Ethical approval was obtained from the local ethics committee ‘Ethikkommission Nordwest- und Zentralschweiz’. All adolescents, their parents and the therapists provided their informed consent.

### Patient sample

This study is part of the multi-center study ‘Evaluation of Adolescent Identity Treatment’ [28, 30] that has been registered at clinicaltrials.gov (NCT02518906). The study aimed at showing non-inferiority of Adolescent Identity Treatment vs Dialectic Behavior Therapy as the outcome research part but, additionally, aims at answering a number of psychotherapy-process questions (see [28]for details). The current analyses are based on the entire available data collected at one participating center (Psychiatric Hospitals of the University of Basel, UPK). The patients were recruited between September 2015 and September 2019 from a specialized consultation for patients with personality disorders which is part of the UPK.

**Fig. 1:**
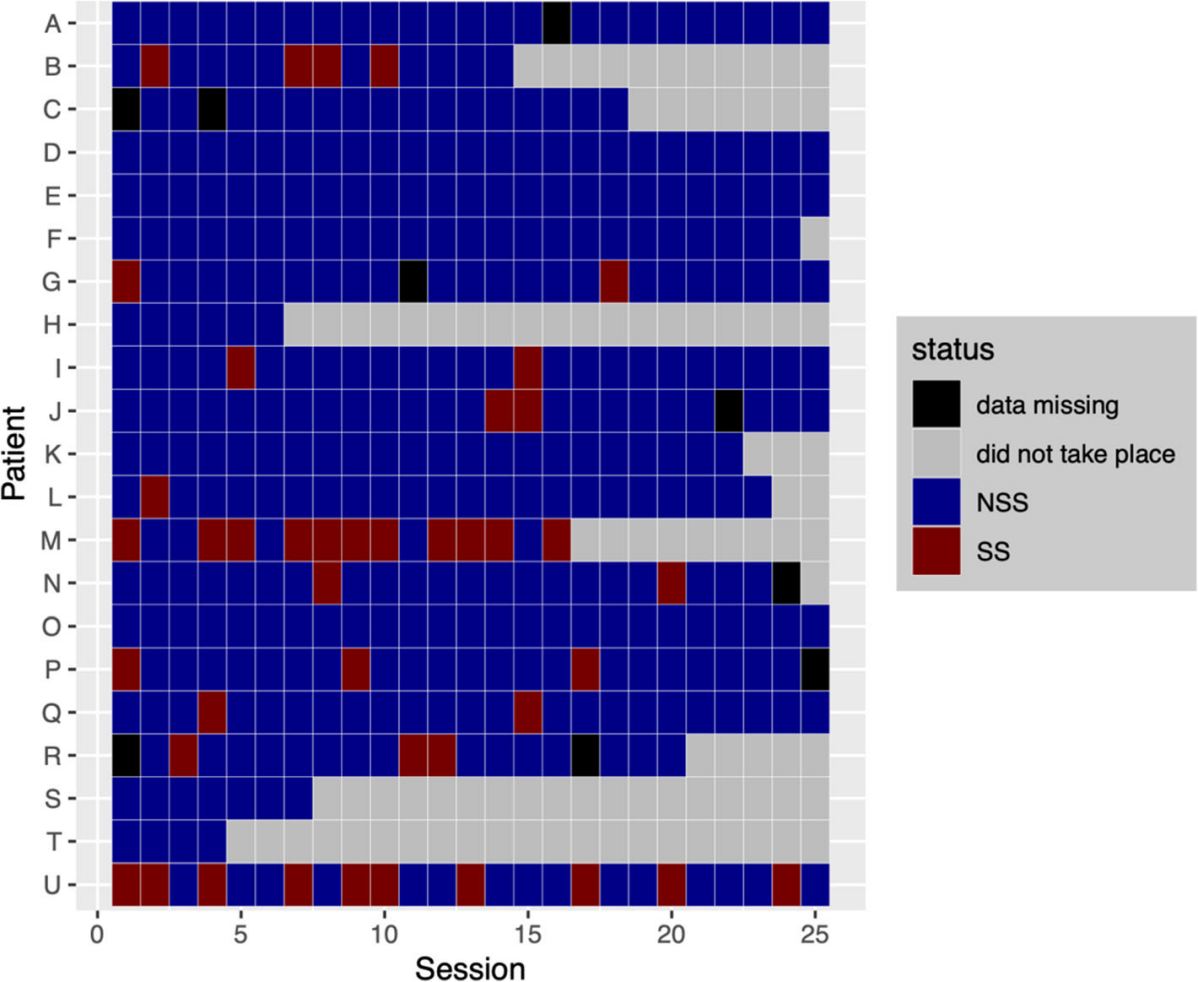
Sessions with and without suicidality addressed. SS = sessions in which suicidality was addressed; NSS = sessions in which suicidality was not addressed. There was a total of 42 SS sessions. *N* = 11 patients had at least one SS session, *N* = 10 did not have any SS session

A total of *N* = 23 adolescents (*N* = 21 female, *N* = 2 male) participated in this study. For each psychotherapy 25 sessions of Adolescent Identity Treatment were planned (AIT; [31]). To increase sample homogeneity, male participants were excluded from data analysis: In woman, deliberate self-harm is often used to communicate distress or to modify other peoples’ behavior whereas in males deliberate self-harm is associated with greater suicidal intent [32]. Moreover, suicidal ideation is reported far more often by woman than by men. For this reason, the two male patients could be outliers. The following inclusion criteria were applied for the patients: age 13–19 years; three or more BPD criteria (Structured Clinical Interview for DSM-IV Axis II Personality Disorders [SCID-II] [33];); and identity diffusion according to the Assessment of Identity Development in Adolescence (AIDA; total *t* score > 60 [34, 35];). The mean age of the remaining 21 female patients was 16.3 (*SD* = 1.6) years. Fifteen patients presented with BPD and six with sub-threshold-BPD (three or four fulfilled BPD criteria in SKID-II). Six patients dropped out of treatment but were included in this study. Nine recordings of therapeutic sessions were missing due to technical difficulties or human failure. Figure 1 shows the available sessions for each patient.

### Psychotherapy

Eight clinicians were involved in the current study of whom six were female (75%). All therapists were psychologists or psychiatrists who underwent or were currently undergoing psychotherapy training to obtain the Swiss specialist degree “Fachpsychologe/in für Psychotherapie FSP” or "Facharzt für Psychiatrie und Psychotherapie FMH". They were either focused on psychodynamic or systemic psychotherapeutic approaches for children and adolescent. Additionally, the therapists underwent training and supervision in Adolescent Identity Treatment which is a manualized therapeutic approach (AIT [31];). “AIT is a psychodynamic method for the treatment of personality disorders in adolescents and integrates modified elements of Transference-Focused Psychotherapy (TFP [36];) with behavior-oriented home-plans, psychoeducation and a stronger focus on working with parents. The main techniques consist of clarification, confrontation and interpretation focusing on affects in the here and now and on dominant object-relationship dyads [31]. All psychotherapists received specific training for AIT. Adherence and competence of the psychotherapists was checked by one of the authors of the AIT manual.

### Suicidality sessions (SS)

After each session, the clinicians had to note information on essential moments in the last session and on suicidality (“Was current suicidality addressed?”). Sessions are treated as “sessions in which suicidality was addressed” (SS) if suicidal tendency had either been mentioned as an essential moment or if the question on current suicidality had been answered with yes. All the other sessions were labeled as “sessions in which suicidality was not addressed” (NSS). The term suicidality comprises current suicidal ideation, a concrete suicide plan and suicide attempt and excludes non-suicidal self-injury behavior. Figure 1 shows SS and NSS for each patient.

### Session evaluation questionnaire (SEQ)

Therapist response was measured using the Session Evaluation Questionnaire (SEQ; [37–39]), Form 5. It comprises 21 seven-point bipolar adjective scales on which therapists and patients are instructed to circle the appropriate number to show how they perceived the previous session. The items are divided into two sectors: Session evaluation (“This session was …” ) and post-session mood (“Right now I feel...”). Post-session mood included: happy-sad, angry-pleased, moving-still, uncertain-definite, calm-excited, confident-afraid, friendly-unfriendly, slow-fast, energetic-peaceful and quiet-aroused. Every item can either be subsumed under the subscale “positivity” or “arousal”. Internal consistency, measured by coefficient alpha, was high for all SEQ indexes across different settings and conditions [39]. Of particular interest for this study were the items calm-excited and quiet-aroused from the subscale “arousal”, and the items confident-afraid, angry-pleased and uncertain-definite from the subscale “positivity”. The German version (SEQ-D) was used which presents with good psychometric properties [40].

### Statistical analyses

Data analysis was performed with R [41]. Principal component analysis (PCA) was used to reduce the dimensionality of the five therapist response variables (angry-pleased, calm-exciting, confident-afraid, quiet-aroused, uncertain-definite). PCA performs this task by creating new uncorrelated variables that successively maximize variance. The result is defined by the data at hand and not a priori [29, 42]. A scree plot was used to select relevant principal components. For hypothesis testing, a linear mixed-effect model with random intercept was used (R package ‘nlme’ [43]) to predict each relevant principal component of the therapist responses in the Session Evaluation Questionnaire (SEQ). Data about SS vs NSS was used as the dependent variable. The patient-therapist dyad was used as the grouping factor for the random effects. For statistical significance, alpha was set to 0.05. Model assumptions were verified using Q-Q- and residual-plots.

## Results

### Sample

*N* = 11 patients (52%) have talked in at least one session about suicidal ideation, the wish to kill themselves or about a concrete suicide plan. On average, the participating patients (*N* = 21) reported a lifetime suicide attempt history with *M* = 2.86 attempts (*SD* = 6.85). In the sample in which suicidality had been addressed during psychotherapy (*N* = 11), lifetime suicide attempt history measured *M* = 4.82 attempts (*SD* = 9.43), ranging from 0 to 30 attempts. The remaining sample (*N* = 10) had a lifetime suicide attempt history with *M* = 0.70 attempts (*SD* = 0.68) with a range from 0 to 2 attempts.

### Description of sessions in which suicidality has been addressed (SS)

*N* = 42 sessions were labeled as suicidality-sessions (SS; 10%) and *n* = 376 as non-suicidality-sessions (NSS; 90%). From these 11 patients, talking about suicidality appeared in *M* = 20% of the sessions, ranging from 4 to 69%. Five patients (46%) mentioned suicidality twice during psychotherapy, two patients (18%) three times, one patient once, one patient four times, one patient ten and one eleven times (*M* = 3.82, *SD* = 3.24, *Mdn* = 2).

### Principal component analysis

This step was used to extract principal components from the SEQ data as a preparatory step to later correlate the extracted components with observed suicidality (see section “Hypothesis testing” below). The five principal components (PC1, PC2, PC3, PC4, and PC5) extracted from the SEQ-Items (calm – excited, quiet – aroused, confident – afraid, uncertain – definite and angry – pleased) explained 53, 23, 9, 8, and 7% of the variance in the suicidality data. In Table 1, the correlation of the original variables with the principal components is shown. According to the Scree-Test [44] and the Kaiser-Guttman rule [45, 46], two components (PC1 and PC2) were extracted. PC1 explained more than half of the variance (53%). It correlated positively with the items calm – excited, confident – afraid and quiet – aroused, and negatively with the items uncertain – definite and angry – pleased. High PC1 scores involve therapist uncertainty, anger, excitement, fear and arousal. PC2 explained 23% of the variance and correlated positively with the items quiet – aroused, angry – pleased, calm – excited, and uncertain – definite and negatively but negligibly with the item confident – afraid. Higher PC2 scores go along with higher therapist arousal, satisfaction, excitement and definiteness.

**Table 1:** Principal component (PC) loadings

Item	PC1	PC2	PC3	PC4	PC5
calm – excited	0.49	0.36	−0.64	0.47	0.07
quiet – aroused	0.33	0.74	0.55	0.15	−0.12
confident – afraid	0.40	−0.12	−0.05	0.49	0.76
uncertain – definite	- 0.50	0.28	0.20	0.49	0.63
angry – pleased	- 0.49	0.48	−0.50	0.52	−0.04

The table shows the correlation of the extracted principal components (PC1 – PC5) with the SEQ items (calm – excited, quiet – aroused, confident – afraid, uncertain – definite, angry – pleased)

### Description of therapist response according to session evaluation questionnaire (SEQ)

Descriptive data of therapist response measured by the SEQ items is shown in Table 2. The lower a score is the more it corresponds to the left adjective in the bipolar scale, the higher a score is the more it corresponds to the right one. The average score over all items was *M* = 3.37 in SS and *M* = 2.82 in NSS which indicates that on average therapists felt more angry, excited, afraid, uncertain and aroused after SS. PC1- and PC2-scores condense these results.

**Table 2:** Description of therapists' emotional states in the SEQ in SS vs NSS

Description of SEQ in SS					Description of SEQ in NSS					
	Mean	Median	SD	Min	Max	Mean	Median	SD	Min	Max
angry-plea.	4.93	5.00	1.22	3.00	7.00	5.27	6.00	1.47	1.00	7.00
calm-exc	3.50	3.00	1.53	1.00	7.00	2.85	2.00	1.37	1.00	6.00
confident-afr.	2.83	3.00	1.27	1.00	6.00	2.39	2.00	1.13	1.00	6.00
uncertain-def.	4.76	5.00	1.41	2.00	7.00	5.39	6.00	1.34	1.00	7.00
quiet-arou.	4.19	4.50	1.53	2.00	7.00	3.53	3.00	1.39	1.00	7.00
PC1	1.08	0.81	2.49	−3.41	6.49	−0.12	−0.45	2.14	−4.07	6.58
PC2	0.29	0.24	1.34	−2.63	3.05	−0.03	−0.17	1.48	−4.45	4.01

*plea.* pleased, *exc.* excited, *afr.* afraid, *def.* definite, *arou.* aroused

### Hypothesis testing

**Fig. 2:**
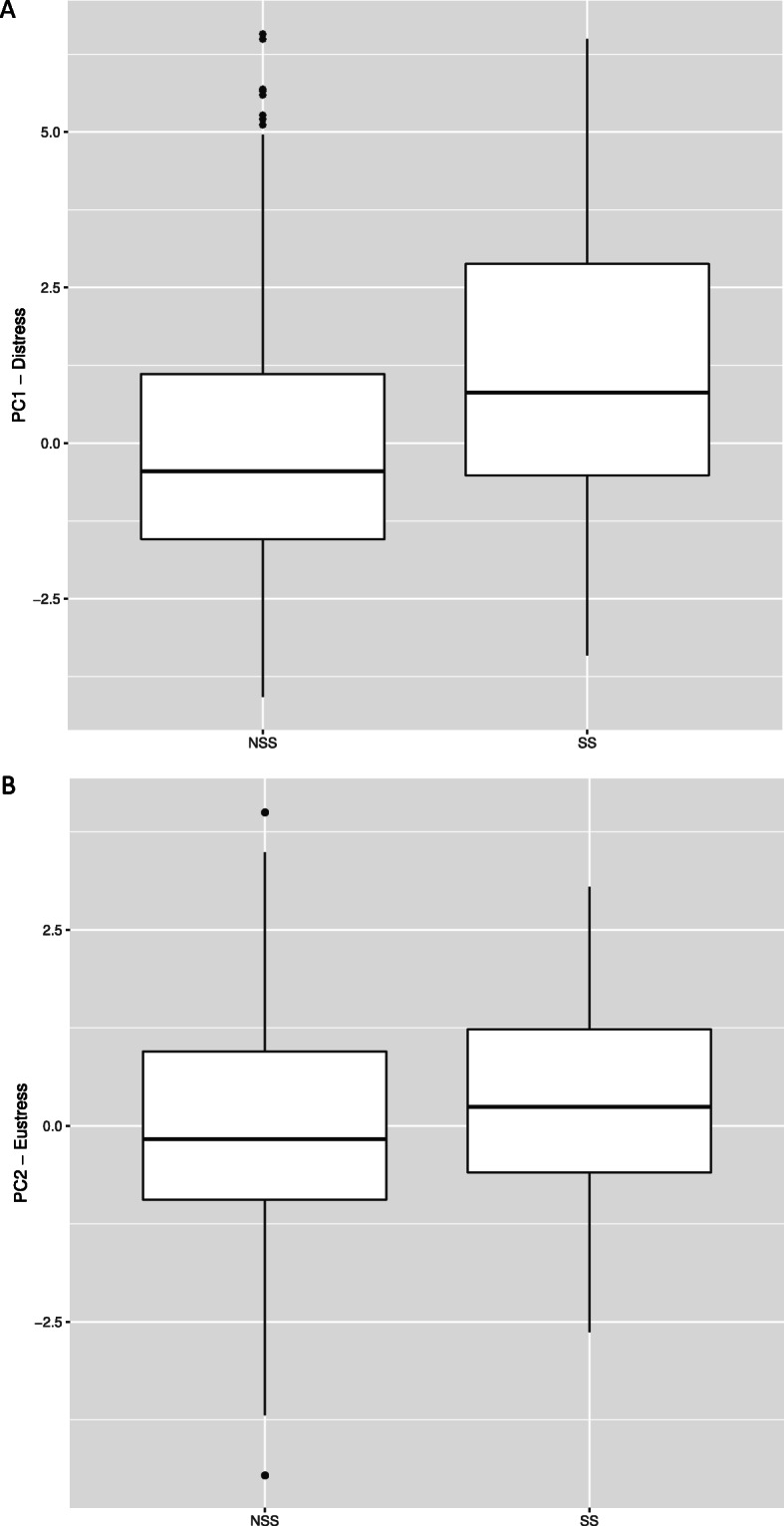
Comparison of therapists' emotional states after sessions with and without suicidality addressed . SS = sessions in which suicidality was addressed; NSS = sessions in which suicidality was not addressed

The linear mixed-effects model showed that addressing suicidality during a session (NSS vs SS) was significantly positively correlated with both the PC1 (b = 0.87, *SE* = 0.36, *t* (418) = 2.40, *p* < 0.05, ICC = 0.19, σ2 = 3.85, deviance = 1784.2) and the PC2 therapist response pattern (b = 0.23, *SE* = 0.24, *t* (418) = 0.95, *p* < 0.001, ICC = 0.18, σ2 = 1.73, deviance = 1450.4). The hypothesis that therapists are more aroused, excited, afraid, uncertain and angry after SS than after NSS (see Fig. 2a), is confirmed. Unexpectedly, the model using PC2 as dependent variable implies another emotional pattern related to suicidality: Therapists are significantly more exited, aroused, definite and pleased after SS than after NSS (see Fig. 2b).

## Discussion

The aim of the current study was to investigate therapists' emotional responses toward young female adolescents with borderline pathology talking about their suicidal ideations or about a concrete suicide plan. We hypothesized that therapists’ post-session emotion would differ depending on whether suicidality came up as a topic or not (SS vs NSS). We expected that therapists are more afraid, excited, aroused, uncertain and angry after SS than after NSS. Therapist response was measured by Session Evaluation Questionnaire (SEQ) which had to be filled out by all therapists after each session. Results showed that SS are accompanied by higher therapist arousal. PC1 reflects that SS are associated with higher clinician excitement, anger, fear, arousal and uncertainty than NSS. Moreover, PC2 explained 23% of the variance in the therapist response items predicted by suicidality. This pattern is also characterized by higher arousal and excitement but demonstrates as well satisfaction and definiteness.

Previous research on this topic has especially dealt with therapists’ emotional responses towards “suicidal patients”. For example, a study with suicidal adolescents has shown that due to therapists' countertransference and due to trait-like suicidal ideation, therapists are unwilling to treat those patients at risk for suicide [47]. In patients with BPD it was suggested that the history of suicide attempts was associated with a worse general level of personality organization [48]. In contrast, the current study underlines the importance of significant psychotherapy moments with regard to therapists’ emotional response and shows that specific episodes and sessions affect therapists. Shifting the focus from “difficult patients” to identifying subjectively difficult or even precarious moments in therapy allows clinicians to seek help on a more tangible level.

PC1 reflects therapists’ negative affect and arousal and is to be summarized in the term “distress”. This is consistent with qualitative research [5, 6] and seems highly associated with patients’ suicidal statements since this component explains more than half of the variance in the SEQ data. Distress is to be understood as the “negative stress response, often involving negative affect and physiological reactivity: a type of stress that results from being overwhelmed by demands, losses or perceived threats. It has a detrimental effect by generating physical and psychological maladaptation and posing serious health risks for individuals. This generally is the intended meaning of the word stress” [1]. The concept of distress [49] emphasizes the importance of feeling able to meet the challenge by appropriate coping strategies. Therapists are often confronted with difficult situations in therapy and have a lot of responsibility. To ensure therapists’ mental health, which is the foundation of their work [50], it is important to strengthen therapists’ coping strategies.

In contrast to this, PC2 seems to point towards a positive kind of arousal whereby therapists feel definite and pleased. PC2 shows similarities with the concept of “eustress” and will be referred to as ‘eustress-component’. In the APA Dictionary of Psychology, eustress is understood as a “positive stress response, involving optimal levels of stimulation: a type of stress that results from challenging but attainable and enjoyable […] tasks […]. It has a beneficial effect by generating a sense of fulfillment or achievement and facilitating growth, development, mastery, and high levels of performance” [1]. This state is not a threat but a challenge and leads to positive feelings if the therapist thinks the situation was well managed. The correlation of SS with the eustress-component is of importance for clinical practice: Patient suicidality does not automatically have to be burdening for therapists and they can even feel more pleased and definite after sessions in which suicidality was addressed compared to sessions in which it was not. Addressing suicidality in psychotherapy can be a window of opportunity.

Of course, causality cannot be inferred from this correlation of patient suicidality and therapists’ emotional state after session in which suicidality is addressed. For example, an alternative hypothesis could be stated that therapists’ emotional state during the session has an influence on the patients in whether or not they raise the issue of suicidality. Another variant could be that the mood of the therapists has nothing to do with the session per se and thus represents a finding by chance. Moreover, Krause & Lutz [51] point out that therapist, patient, and context variables cannot be separated and affect each other. In this respect, it can be assumed that therapists respond to patients, but patients also respond to therapists, which makes any conclusive statement impossible. Thus, the therapeutic situation is a result of responsiveness both of the part of the therapist as of the patient.

### Clinical implications

Although countertransference is a complex concept with a meaningful historical background, it offers a good possibility to do justice to the multifaceted feelings therapists are confronted with during psychotherapy – both consciously and unconsciously. Since working with suicidal patients can be a very challenging and anxiety-provoking task for psychotherapists, their awareness of concrete emotional patterns in sessions where patients seem at risk for suicide is essential and should discourage the temptation of acting out unconscious feelings or conflicts [52]. Avoiding therapist errors emanating from negative countertransference improves the therapeutic relationship and finally also the patient’s suicidal tendency [53]. Thus, supervision and notes on therapy sessions should sensitize clinicians to their emotional reaction for specific sessions where suicidality has come up as a topic.

Secondly, we proposed that addressing suicidal ideation during psychotherapy is associated with both distress and eustress in therapists. The question remains of what distinguishes the therapeutic “distress” reaction from the “eustress” reaction and how the former can be transformed into the latter. In this regard, our results suggest that the main difference between these two emotional responses is that the “eustress” response includes a sense of definiteness and satisfaction and the absence of anxiety, anger and uncertainty which in turn facilitates development and the mastery of a concrete situation. Therefore, it is crucial that therapists feel definite, self-effective and act in the feeling that they are part of the therapy process. By being aware of possible conflictive emotions, a stable therapeutic relationship can be established which in turn leads to a promising outcome. A focus for how therapists can be supported to feel comfortable in challenging situations and a clinical culture that allows for errors and uncertainties should be established. Again, supervision can be helpful for clinicians to gain understanding of a difficult moment in the therapeutic process.

Thirdly, it should be kept in mind that suicidal ideation is not always reason for an alarm and that it can overshadow other important issues that should be discussed in psychotherapy [54]. Accordingly, it is suggested that – although suicidal ideation should always be taken seriously – the amount of focusing on suicidality should be skillfully balanced with the focus on other meaningful topics.

### Limitations

Despite the importance of this topic, some limitations of the current study need to be considered. First of all, the study includes a relatively small sample size of *N* = 21 dyads. Only 11 of these dyads discussed the topic of suicidality. However, in terms of investigated sessions, the sample size was 418 sessions of which 211 sessions stemmed from dyads who discussed suicidality. The sample size in the current study is based on a power analysis which was aimed to sufficiently power an outcome comparison (see study design in [28]). It was difficult to estimate how many of the patients would discuss suicidality during the psychotherapy. In this sense, the current study has an explorative character that can serve as a basis for future studies. A replication of the current results is necessary to confirm the observed effect which was significant despite the small sample size.

Secondly, the patients’ suicidality was not documented with a validated scale. The therapists documented whether suicidality was addressed or not after each session. Our rationale is that the discussion of suicidality is a salient event and determining whether the event occurred or not should be simple and straightforward. However, a validated scale might be helpful in getting more differentiated results to determine, for instance, the perceived intensity of addressing the topic or the topic's actual acuteness for the patient. Thirdly, therapist response was measured with the Session Evaluation Questionnaire (SEQ) which is a self-rating questionnaire that is not constructed as a stress-test. Although this seems adequate for a subjective description of post-session mood, it should be complemented by a validated stress questionnaire and by psychophysiological measures such as saliva cortisol level or electrodermal skin response to measure the reaction at the level of the autonomous nervous system [55–57].

## Conclusion

Two different therapist responses were identified after sessions in which young female patients with BPD or subthreshold BPD talked about their suicidal ideation or about a more concrete suicide plan. Therapists showed an emotional response pattern consisting firstly of arousal and negative affect (“distress”) and secondly of arousal and positive affect (“eustress”). These two types of arousal are distinguished by being angry, uncertain and afraid versus feeling pleased and definite. First of all, this implies that patient suicidality does not always have to be emotionally burdening for therapists and moreover, that clinicians need to feel definite and self-effective which in turn enables the mastery of a challenging situation. If therapists become aware of their “suicidality-countertransference” and their anxiety about patient suicide contained, this can on one hand enable a good therapy process and outcome (including prevent discontinuation of the therapeutic process) and on the other hand lead to clinicians’ well-being. Conducting further research on how therapists’ mental health can be preserved seems necessary in order to ensure appropriate and good care for patients with suicidal tendencies.

## Data Availability

The datasets during and/or analysed during the current study available from the corresponding author on reasonable request.

## References

[1] VandenBos GR (2015). APA dictionary of psychology.

[2] Ellis TE, Schwartz JAJ, Rufino KA (2018). Negative reactions of therapists working with suicidal patients : a CBT / mindfulness perspective on “Countertransference”. J Cogn Ther.

[3] Kleespies PM, Penk WE, Forsyth JP (1993). The stress of patient suicidal behavior during clinical training: incidence, impact, and recovery. Prof Psychol Res Pract.

[4] Schmitz WM, Allen MH, Feldman BN (2012). Preventing suicide through improved training in suicide risk assessment and care: an american association of suicidology task force report addressing serious gaps in U.S. mental health training. Suicide Life Threat Behav.

[5] Deutsch CJ (1984). Self-reported sources of stress among psychotherapists. Prof Psychol Res Pract.

[6] Farber BA (1983). Psychotherapists’ perceptions of stressful patient behavior. Prof Psychol Res Pract.

[7] Goodman M, Roiff T, Oakes AH, Paris J (2012). Suicidal risk and management in borderline personality disorder. Curr Psychiatry Rep.

[8] American Psychiatric Association Practice Guidelines (2001). Practice guideline for the treatment of patients with borderline personality disorder. American Psychiatric Association. Am J Psychiatry.

[9] Chanen A, Sharp C, Hoffman P (2017). Prevention and early intervention for borderline personality disorder: a novel public health priority. World Psychiatry.

[10] World Health Organization (2018). International classification of diseases for mortality and morbidity statistics (11th Revision).

[11] Birkhölzer M, Schlüter-Müller S, Schmeck K (2020). Persönlichkeitsstörungen im Kindes- und Jugendalter. Swiss Arch Neurol Psychiatry Psychother.

[12] Barzilay S, Yaseen ZS, Hawes M, Gorman B, Altman R, Foster A, et al. Emotional responses to suicidal patients: factor structure, construct, and predictive validity of the therapist response questionnaire-suicide form. Front Psychiatry. 2018;9 10.3389/fpsyt.2018.00104. 10.3389/fpsyt.2018.00104 PMC5895710 29674979

[13] Kernberg O (1965). Notes on countertransference. J Am Psychoanal Assoc.

[14] Maltsberger JT, Buie DH (1974). Countertransference hate in the treatment of suicidal patients. Arch Gen Psychiatry.

[15] Yaseen ZS, Briggs J, Kopeykina I, Orchard KM, Silberlicht J, Bhingradia H, Galynker II (2013). Distinctive emotional responses of clinicians to suicide-attempting patients - a comparative study. BMC Psychiatry.

[16] Fowler JC (2013). Core principles in treating suicidal patients. Psychotherapy.

[17] Allen JG, Jobes DA, Michel K (2011). Mentalizing suicidal states. Building a therapeutic alliance with the suicidal patient.

[18] Pope KS, Tabachnick BG (1993). Therapists’ anger, hate, fear, and sexual feelings: national survey of therapist responses, client characteristics, critical events, formal complaints, and training. Prof Psychol Res Pract.

[19] Hendin H, Haas AP, Maltsberger JT, Koestner B, Szanto K (2006). Problems in psychotherapy with suicidal patients. Am J Psychiatry.

[20] Miller G, Iverson K, Kemmelmeier M (2011). A preliminary examination of burnout among counselor trainees treating clients with recent suicidal ideation and borderline traits. Couns Educ Superv.

[21] Richards BM (2000). Impact upon therapy and the therapist when working with suicidal patients: some transference and countertransference aspects. Br J Guid Couns.

[22] Michaud L, Greenway KT, Corbeil S, Bourquin C, Richard-Devantoy S. Countertransference towards suicidal patients: a systematic review. Curr Psychol. 2021; 10.1007/s12144-021-01424-0.

[23] Dressler DM, Prusoff B, Mark H, Shapiro D (1975). Clinician attitudes toward the suicide attempter. J Nerv Ment Dis.

[24] Yaseen ZS, Galynker II, Cohen LJ, Briggs J (2017). Clinicians’ conflicting emotional responses to high suicide-risk patients—association with short-term suicide behaviors: a prospective pilot study. Compr Psychiatry.

[25] Colson DB, Allen JG, Coyne L, Dexter N, Jehl N, Mayer CA, Spohn H (1986). An anatomy of countertransference: staff reactions to difficult psychiatric hospital patients. Hosp Community Psychiatry.

[26] Gillig PM, Hillard JR, Deddens JA, Bell J, Combs HE (1990). Clinicians’ self-reported reactions to psychiatric emergency patients: effect on treatment decisions. Psychiatr Q.

[27] Chui H, Hill CE, Kline K, Kuo P, Mohr JJ (2016). Are you in the mood? Therapist affect and psychotherapy process. J Couns Psychol.

[28] Zimmermann R, Krause M, Weise S, Schenk N, Fürer L, Schrobildgen C, Schlüter-Müller S, Valdes N, Koenig J, Kaess M, Schmeck K (2018). A design for process-outcome psychotherapy research in adolescents with borderline personality pathology. Contemp Clin Trials Commun.

[29] Zimmermann R, Fürer L, Schenk N, Koenig J, Roth V, Schlüter-Müller S, Kaess M, Schmeck K (2020). Silence in the psychotherapy of adolescents with borderline personality pathology. Personal Disord Theory Res Treat.

[30] Schmeck K, Pick OG, Milidou M (2018). Früherkennung von Persönlichkeitsstörungen. PTT - Persönlichkeitsstörungen Theor und Ther.

[31] Foelsch PA, Schlüter-Müller S, Odom AE, et al. Adolescent identity treatment. Switzerland: Springer International Publishing Switzerland; 2014. 10.1007/978-3-319-06868-8 / https://link.springer.com/book/10.1007/978-3-319-06868-8#about.

[32] Hawton K (2000). Sex and suicide: gender differences in suicidal behaviour. Br J Psychiatry.

[33] First MB, Gibbon M, Spitzer RL (1997). Structured clinical interview for DSM-IV axis II personality disorders (SCID-II).

[34] Goth K, Foelsch P, Schlüter-Müller S, Birkhölzer M, Jung E, Pick O, Schmeck K (2012). Assessment of identity development and identity diffusion in adolescence - theoretical basis and psychometric properties of the self-report questionnaire AIDA. Child Adolesc Psychiatry Ment Health.

[35] Lind M, Vanwoerden S, Penner F, Sharp C (2019). Inpatient adolescents with borderline personality disorder features: identity diffusion and narrative incoherence. Personal Disord Theory Res Treat.

[36] Kernberg O, Yeomans F, Clarkin J, Levy K (2008). Transference focused psychotherapy: overview and update. Int J Psychoanal.

[37] Stiles WB (1980). Measurement of the impact of psychotherapy sessions. J Consult Clin Psychol.

[38] Stiles WB, Snow JS (1984). Counseling session impact as viewed by novice counselors and their clients. J Couns Psychol.

[39] Stiles WB (2000). Session evaluation questionnaire: structure and use.

[40] Hartmann A, Leonhart R, Hermann S, Joos A, Stiles WB, Almut Zeeck (2013). Die Evaluation von Therapiesitzungen durch Patienten und Therapeuten: Faktorstruktur und Interpretation des SEQ-D. Diagnostica.

[41] R Core Team (2018). R: a language and environment for statistical computing [Computer software].

[42] Jollife IT, Cadima J (2016). Principal component analysis: a review and recent developments. Philos Trans R Soc A Math Phys Eng Sci.

[43] Pinheiro J, Bates D, DebRoy S (2018). nlme: linear and nonlinear mixed effects models [computer software].

[44] Cattell RB (1966). The scree test for the number of factors. Multivar Behav Res.

[45] Guttman L. A new approach to factor analysis: the Radex. In: Lazarsfeld PF, editor. Mathematical thinking in the social sciences: Free Press; 1954. p. 258–348. https://psycnet.apa.org/record/1955-02329-001.

[46] Kaiser HF (1960). The application of electronic computers to factor analysis. Educ Psychol Meas.

[47] Gvion Y, Rozett H, Stern T (2020). Will you agree to treat a suicidal adolescent? A comparative study among mental health professionals. Eur Child Adolesc Psychiatry.

[48] Baus N, Fischer-Kern M, Naderer A, Klein J, Doering S, Pastner B, Leithner-Dziubas K, Plener PL, Kapusta ND (2014). Personality organization in borderline patients with a history of suicide attempts. Psychiatry Res.

[49] Selye H, Serban G (1976). Stress without distress. Psychopathology of human adaptation.

[50] Sherman MD (1996). Distress and professional impairment due to mental health problems among psychotherapists. Clin Psychol Rev.

[51] Krause MS, Lutz W (2009). Process transforms inputs to determine outcomes: therapists are responsible for managing process. Clin Psychol Sci Pract.

[52] Gelso CJ, Latts MG, Gomez MJ, Fassinger RE (2002). Countertransference management and therapy outcome: an initial evaluation. J Clin Psychol.

[53] Perry JC, Bond M, Presniak MD (2013). Alliance, reactions to treatment, and countertransference in the process of recovery from suicidal phenomena in long-term dynamic psychotherapy. Psychother Res.

[54] Podlogar T, Poštuvan V, De Leo D, Žvelc G (2020). The model of dynamic balance in therapists’ experiences and views on working with suicidal clients: a qualitative study. Clin Psychol Psychother.

[55] Nater U, Rohleder N, Gaab J (2005). Human salivary alpha-amylase reactivity in a psychosocial stress paradigm. Int J Psychophysiol.

[56] Kirschbaum C, Hellhammer DH (1994). Salivary cortisol in psy- choneuroendocrine research: recent developments and applications. Psychoneuroendocrinology.

[57] Chrousos GP, Gold PW (1992). The concept of stress and stress system disorder. An overview of physical and behavioral homeostasis. J Am Med Assoc.

